# De novo variant of *TRRAP* in a patient with very early onset psychosis in the context of non-verbal learning disability and obsessive-compulsive disorder: a case report

**DOI:** 10.1186/s12881-018-0711-9

**Published:** 2018-11-13

**Authors:** Chrystal F. Mavros, Catherine A. Brownstein, Roshni Thyagrajan, Casie A. Genetti, Sahil Tembulkar, Kelsey Graber, Quinn Murphy, Kristin Cabral, Grace E. VanNoy, Matthew Bainbridge, Jiahai Shi, Pankaj B. Agrawal, Alan H. Beggs, Eugene D’Angelo, Joseph Gonzalez-Heydrich

**Affiliations:** 1 Division of Genetics and Genomics, Boston Children’s Hospital, Harvard Medical School, 3 Blackfan Circle CLS 16009, 300 Longwood Avenue, Boston, MA 02115 USA; 2 The Manton Center for Orphan Disease Research, Boston Children’s Hospital, Harvard Medical School, 3 Blackfan Circle, CLSB 15031, Boston, MA 02115 USA; 3 Developmental Neuropsychiatry Program, Department of Psychiatry, Boston Children’s Hospital, Harvard Medical School, 300 Longwood Avenue, Boston, MA 02115 USA; 4 Codified Genomics, 3507 Mosley Court Unit H, Houston, TX USA; 5 Department of Biomedical Sciences, City University of Hong Kong, 1/F, Block 1, To Yuen Building, 31 To Yuen Street, Kowloon Tong, Hong Kong

**Keywords:** Psychosis, Obsessive compulsive disorder, Childhood onset psychosis, Very early onset psychosis, Major depression with psychotic features

## Abstract

**Background:**

*TRRAP* encodes a multidomain protein kinase that works as a genetic cofactor to influence DNA methylation patterns, DNA damage repair, and chromatin remodeling. TRRAP protein is vital to early neural developmental processes, and variants in this gene have been associated with schizophrenia and childhood disintegrative disorder.

**Case presentation:**

Here, we report on a patient with a de novo nonsynonymous *TRRAP* single-nucleotide variant (EST00000355540.3:c.5957G > A, p.Arg1986Gln) and early onset major depression accompanied by a psychotic episode (before age 10) that occurred in the context of longer standing nonverbal learning disability and a past history of obsessions and compulsions.

**Conclusions:**

The de novo variant and presentation of very early onset psychosis indicate a rare Mendelian disorder inheritance model. The genotype and behavioral abnormalities of this patient are reviewed.

**Electronic supplementary material:**

The online version of this article (10.1186/s12881-018-0711-9) contains supplementary material, which is available to authorized users.

## Background

Very early onset psychosis (VEOP) is defined as psychosis with onset prior to age 13 years. Some children with VEOP will ultimately be found to have very early onset schizophrenia, which is rare, affecting approximately 1 in 40,000 children [1]. Others will ultimately be determined to have an affective psychosis, usually major depression or bipolar disorder with psychotic features. Psychosis also occurs in a significant percentage of children under 13 years who suffer from bipolar disorder and major depression; it is difficult early in the course of VEOP to reliably predict whether a child will go on to have schizophrenia (SZ) or an affective psychosis [2–4]. Additionally there is increasing biological evidence for considerable overlap between genes associated with SZ and those associated with affective psychosis such as bipolar disorder [5]. As this genetic overlap between SZ and affective psychosis argues that their pathophysiology is likely to overlap, the National Institute of Mental Health (NIMH) has encouraged the study of psychotic symptoms across diagnostic groups [6].

Although not associated with an OMIM disorder, de novo variants in the transformation/transcription domain associated protein gene (*TRRAP,* located at 7q22.1, NM_003496, OMIM gene description *603015) have been identified in neurological disorders. De novo variants have been reported in epilepsy/epileptic encephalopathy, schizophrenia, and childhood disintegrative disorder (CDD) [7].

TRRAP is a large, multi-domain protein kinase that works as a cofactor in cells to help mediate histone acetylation. Broadly expressed in many tissues of the body and in the brain, the gene is also involved in DNA damage repair and chromatin remodeling [8–10]. When compared with murine *Trrap*+/+ and *Trrap*+/− genotypes, *Trrap*−/− blastocyst embryos at E3.5 show severe growth retardation of the trophoblast layer and the absence of an inner cell mass [11]. Mice with *Trrap* deletion in the CNS develop to term but die at birth. Histological examination of the newborn brains (embryonic day 18.5) revealed severe atrophy, with large ventricular cavities and a pronounced decrease in thickness and disorganized layers in the newborn cortex, indicating that TRRAP is expressed in the prenatal cortex [12].

TRRAP works by recruiting the coactivator complex histone acetyltransferase (HAT) and guiding it to transcription factors bound in chromatin. HAT will then deacetylate the histone proteins surrounding the DNA, allowing select genes to be transcribed.

Inhibition of histone deacetylation has been strongly linked to neuronal differentiation: both in mouse embryonic neural stem cells [13] and in adult rat neural progenitor cells [14]. Even though the precise model for how histone acetylation affects the differentiation of neural stem cells is unknown, links between *TRRAP* and neural proliferation have been made. Studies have shown that deletion of *TRRAP* in cells leads to longer cell cycles and premature differentiation [12, 15–17].

Studies of various gene manipulations, including *TRRAP*, have illustrated that premature differentiation of neurons and abnormal neurogenesis alter neural networks and can affect functionality [12, 15, 16]. Aberrant neuronal development has been associated with schizophrenia and other forms of psychosis [17, 18].

A *TRRAP* de novo mutation has been reported in a study of de novo mutations in schizophrenia [19]. The same research group also showed that *TRRAP* was one of four genes (*LAMA2*, *DPYD*, *TRRAP* and *VPS39*) that were affected by de novo variants a significant number of times in a sample population of 231 subjects with schizophrenia [20]. This study classified *TRRAP* as a prenatally-biased gene, meaning it is highly expressed during first and second trimester [20]. De novo variants in prenatally-biased genes have been linked to an increased likelihood of having multiple behavioral abnormalities in childhood and worse functional outcome following disease onset [20].

Here we describe a boy with a non-verbal learning disability (NVLD) and a history of obsessive-compulsive disorder (OCD) who presented at the age of 9 years with paranoid delusions of being watched and targeted by unknown persons wanting to kill him. This delusion reached psychotic intensity for several months. His symptoms arose in the context of a major depressive episode and ultimately responded to treatment with fluoxetine. Thus, the best fitting DSM-5 diagnosis [21] is very early onset major depressive disorder with psychotic features. Whole exome sequencing of the patient revealed a novel de novo variant in *TRRAP*. The association of *TRRAP* with numerous neurological conditions, including schizophrenia and CDD, leads us to believe the de novo *TRRAP* variant is responsible for elements of this patient’s early onset psychosis.

## Case report

The proband was born at 38 weeks by emergent cesarean section due to fetal distress but had no further complications. He had some gross and fine motor delays but otherwise his developmental milestones were met on time. He has a history of left amblyopia and mild asthma. He has never had any head injury or loss of consciousness.

The proband was first evaluated by a neurologist at age 5 years due to concerns about school performance. This evaluation revealed above average intelligence, but also showed delays in gross motor skills, and some behavioral concerns. At age 7 years, there were concerns for an attention deficit disorder and patterned behaviors, including compulsive handwashing, licking rituals, and repetitive language. These later decreased in severity.

At age 9, the proband was referred by his outpatient psychotherapist for evaluation of paranoia and possible hallucinations including concerns raised at school regarding the content of his written assignments, such as fear of being hunted and of his mortality. The proband described hearing voices and fear that they may be listening. Additionally, the proband expressed fear that unknown persons were hunting him. Reportedly through discussion, he could be convinced that these thoughts were not real, but episodically his paranoia would worsen.

The proband reported hearing voices as early as age 7 years, but first disclosed this after being confronted at age 9 and did not want to admit he heard voices, citing what others might think. He had a history of becoming aggressive towards peers due to misperceiving neutral actions as being hostile. Over the duration of these symptoms, he also had a period of sustained low mood and expressed thoughts of self-harm.

A neuropsychological evaluation performed at 9 years reported the following: Full scale IQ of 113 with no significant difference between verbal and non-verbal reasoning, but deficits in visual-motor integration, recall for organizational schemes, obsessive/inflexible thinking patterns, and mild social delays consistent with a diagnosis of NVLD. The proband was also noted to have difficulties reading social cues, making friends, deescalating conflicts, and organizing work and integrating details into a larger meaning. Following this evaluation, the proband was diagnosed with major depression with psychotic features in the context of longer standing NVLD and OCD.

Currently, at the age of 11 years, the proband tends to have a few OCD-related transient rituals that have not been debilitating. He has no history of tics. Family history is significant only for maternal anxiety and a maternal great-great-grandmother who suffered from schizophrenia.

The proband began treatment with Fluoxetine at 10 years and 2 months weighing 28.9 kg. This medication was chosen as there is existing literature suggesting that a selective serotonin reuptake inhibitor (SSRI) can be successful when used as monotherapy for delusional depression [22, 23] and fluoxetine was the only SSRI with FDA approval for treatment of depression in his age group [24]. He initially responded to 6 mg per day (1.5 ml of the 20 mg/5 ml formulation), but it was subsequently increased to 10 mg per day (2.5 ml of the 20 mg/5 ml formulation). After 20 months, treatment has appeared to have almost completely eliminated the auditory hallucinations, paranoia, and depression. His anxiety has also improved. However, there have been multiple episodes of thoughts of self-harm during stressful situations and at least one episode of recurrence of auditory hallucinations, leading to increases in fluoxetine dose gradually to 16 mg per day. He is currently being monitored by his treatment team for any return of depression and psychotic symptoms.

### Whole exome sequencing, genetic analysis, and protein modeling

Whole exome sequencing (WES) on the proband and both parents was provided by the Yale Center for Mendelian Genomics. Whole-exome libraries were prepared using KAPA Hyper kit, exome enriched with IDT’s xGen Exome Research Panel v1.0, using multiplexed capture of 16 samples and sequenced on HiSeq4000 using paired-end chemistry at a read length of 100 bp. FASTQs were aligned by Codified Genomics (proprietary algorithm, Houston, TX) (Table 1). The Exome Variant Server, 1000 Genomes, and ClinVar databases were checked on March 21, 2018.

**Table 1:** NGS quality report summary. Rare variants and X-linked variants are defined as having allele frequencies < 1% in ESP5000 (from NHLBI EVS), 1000G and CG52. Compound heterozygous variants are restricted to non-synonymous variants shared heterozygous with each parent and with allele frequencies of < 1% in each reference database. De novo events are defined as all variants in the IDT’s xGen Exome Research Panel target region which are seen in neither parent and are absent from dbSNP, EVS5000, 1000G and were required to have genotype qualities (GATK) > = 20 and read depth > = 10

WES Parameters^a^	Proband	Father	Mother
Captured target size	39 Mb	39 Mb	39 Mb
% target covered by 10+ reads	92.2%	96%	93.2%
Mean read depth of target region	85.6X	110X	89.2X
Total number of SNPs	47,769	50,336	48,307
Total number of INDELs	4651	4733	4642
N rare variants	1018	1008	932
N compound heterozygous variants	22 (11 genes)	N/A	N/A
N X-linked	10		
N de novo events^b^	11	N/A	N/A

^a^Values are limited to variants mapping to the IDT’s xGen Exome Research Panel v1.0 target region

^b^Additional de novo events are listed in Additional file 1: Table S1

Review of the WES data confirmed the reported genetic relationships of the proband and his parents and revealed two single nucleotide variants (SNVs) in the proband with predicted pathogenicity according to at least one in silico prediction program (Table 2). These SNVs occur in genes that are hypothesized to be associated with the phenotype based on current knowledge of gene function, pathway, expression pattern, etc. One is a heterozygous de novo missense variant of *TRRAP*, EST00000355540.3:c.5957G > A, p.Arg1986Gln, and the other is a maternally inherited hemizygous missense variant in the X-linked androgen receptor gene, *AR* (located at Xq12, OMIM gene description * 313700), reported in in ClinVar with “conflicting interpretations of pathogenicity” (rs201934623). Both variants have been confirmed by Sanger sequencing in the trio. Secondary findings unrelated to the phenotype have not been reported.

**Table 2:** *TRRAP* and *AR* Variant Analysis

Chromosomal location	7q22.1	Xq12
Position (GRCh37/hg19)	98,553,863	66,766,162
Gene Name	*TRRAP*	*AR*
Reference	G	C
Number of reads with reference in PROBAND	56	0
Alternative in PROBAND	A	T
Number of reads with alternative in PROBAND	71	39
Number of reads with reference in MOTHER	93	41
Alternative in MOTHER	None	T
Number of reads with alternative in MOTHER	None	62
Number of reads with reference in FATHER	147	50
Alternative in FATHER	None	none
Number of reads with alternative in FATHER	None	none
Variant type	Nonsynonymous SNV	Nonsynonymous SNV
Refseq ID	NM_003496	NM_000044.4
Variant DNA (HGVS nomenclature _c)	c.5957G > A,	c.1174C > T
Variant DNA (HGVS nomenclature _p)	p.Arg1986Gln	p.Pro392Ser
Prediction from SIFT, Polyphen2^a^	SIFT 0.015 (damaging) Polyphen2 D	SIFT 0.005 (damaging) Polyphen2 B

^a^ SIFT and PolyPhen2 scores are derived from Liu et al., 2011. SIFT scores <= 0.05 are predicted “damaging” and > 0.05 are predicted to be “tolerated”. Polyphen2 scores > = 0.2 and < 0.85 are considered “possibly damaging or P” and > = 0.85 are predicted to be “probably damaging or D”

The patient also underwent phlebotomy for chromosomal microarray analysis of peripheral blood lymphocytes. Genomic DNA was examined by array-based comparative genomic hybridization (aCGH) using the ClariView Array (Claritas Genomics, Cambridge, MA). The array contains DNA oligonucleotide probes in or flanking most exons of the evaluated genes. The array is designed to detect most single-exon deletions and duplications. Probe sequences and locations are based on Genome Reference Consortium build 37 (GRCh37)/UCSC hg19. Data analysis was performed with Agilent Genomic Workbench software, using gene-specific filtering by Cartagenia BENCH software (Cambridge, MA). Confirmations of deletions or duplications were performed by MLPA, qPCR, or aCGH. No Copy Number Variations (CNVs) or Loss of Heterozygosity (LOH) were detected.

Protein structure modeling was prepared by PYMOL. The yeast homologue of TRRAP, Tra1 (5OEJ), was chosen as a template to model TRRAP, as Tra1 is matched at position Arg2004 to Arg1986 in human TRRAP. Protein structure was not determined for the androgen receptor variant due to lack of evidence for pathogenicity. Additional de novo variants are listed in Additional file 1: Table S1. According to ACMG classifications, all of them are VUS, as they have one strong piece of evidence in favor of pathogenicity (de novo w/ confirmed parentage), and one or more factors favoring a score of “Benign” (depending on allele frequencies, predicted effects on transcripts, etc).

## Discussion & Conclusions

Here we report two noteworthy variants in the genes *TRRAP* and *AR* in association with early onset psychosis in an 11-year-old boy. The p.Pro392Ser variant in the androgen receptor gene, *AR* (rs201934623), listed with “conflicting interpretations of pathogenicity” in ClinVar due to occurrence in a patient with a Charcot-Marie-Tooth peripheral neuropathy [25], is unlikely to be causative of this condition. This is largely due to a reported incidence of 674/162861 in gnomAD and 317 hemizgyous males (as of April 6, 2018). Taking this observation into consideration, reevaluation of ACMG/AMP 2015 criteria for variant interpretation leads to a revised assessment of “likely benign” for this variant.

**Fig. 1:**
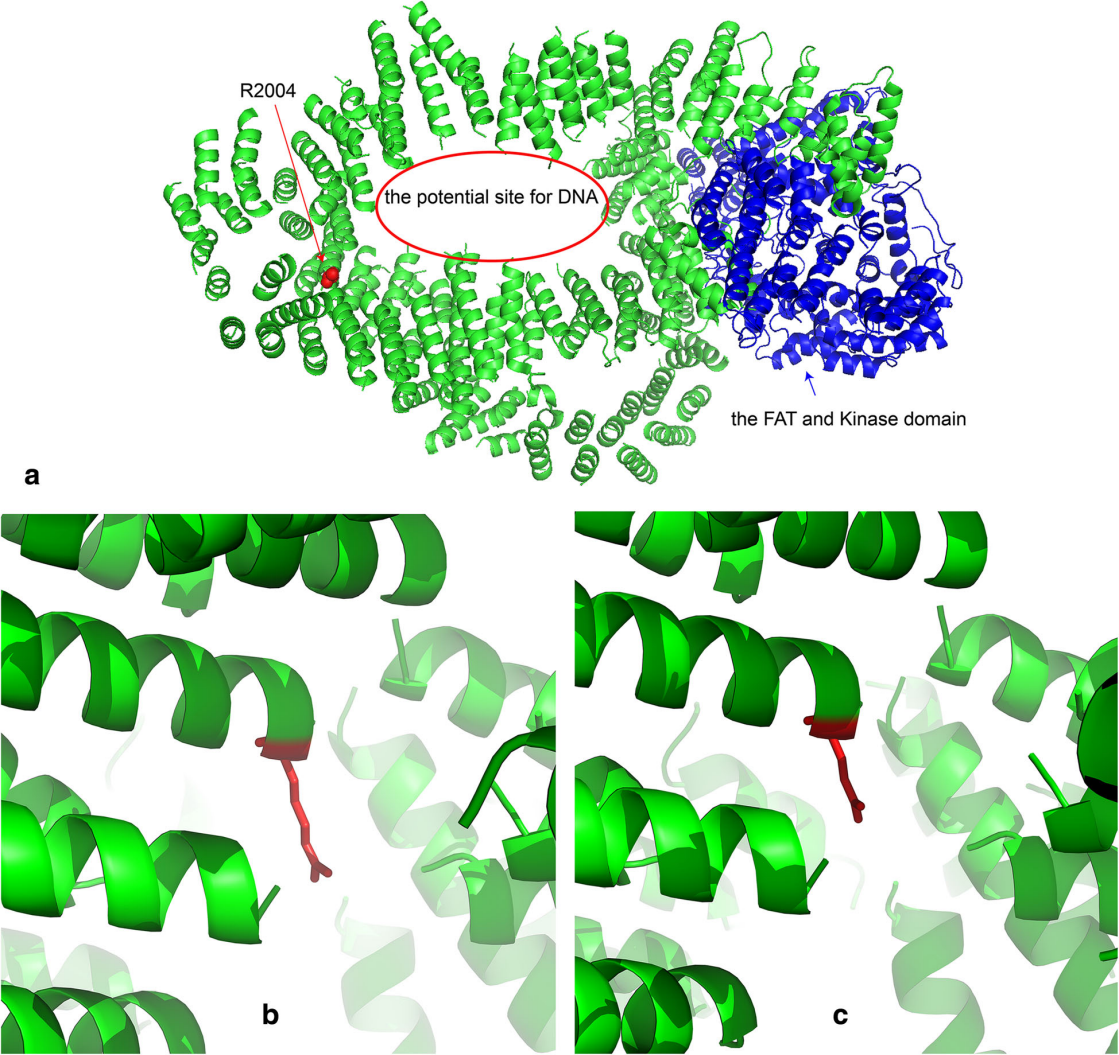
Modeling of the Arg1986 residue in TRRAP. **a** The yeast homologue of TRRAP, Tra1(5OEJ), was chosen as a template to model TRRAP, as Tra1 is matched at position Arg2004 to Arg1986 in human TRRAP. The variant is removed from the kinase domain (noted in blue), and close to the central cavity. **b** Wild-type Arg1986. **c** Arg1986Gln. This substitution may reduce the side chain volume and decrease the binding between TRRAP and DNA

In contrast, available evidence for the de novo *TRRAP* variant, EST00000355540.3:c.5957G > A, p.Arg1986Gln, is consistent with pathogenicity, supporting the possibility that this is causative for elements of the proband’s early onset psychosis (Figure 1). *TRRAP* is a highly conserved gene and extremely intolerant to change, with a pLI score in ExAC of 1.0, indicating almost complete intolerance to loss of function mutations, and a z score of 9.82 for missense variants, indicative of intolerance to variation [26]. Various de novo missense variants of *TRRAP* have been reported in a range of neurological and psychiatric conditions, including epilepsy/epileptic encephalopathy, schizophrenia, and CDD [7]. If one accepts our proposed association of *TRRAP* with early onset psychosis and OCD, then evaluation according to ACMG/AMP 2015 criteria [27] scores the p.Arg1986Gln variant as “likely pathogenic” based on de novo occurrence with confirmed parentage (PS2), and absence from controls in the Exome Sequencing Project, 1000 Genomes, ExAC, and gnomAD databases (PM2). However, a limitation of this study is that WES and not whole genome sequencing was performed, and additional candidate genomic variants outside the exomic capture region may have gone undetected.

Modeling of the Arg1986 residue in TRRAP reveals that it is removed from the kinase domain (noted in blue in Figure 1), and close to the central cavity, which is assumed to be the binding place for DNA (Figure 1a). The Arg1986Gln substitution may reduce the side chain volume and decrease the binding between TRRAP and DNA. (Figures 1b (wt) and 1c (mut)).

A *TRRAP* p.Pro1781Ser de novo variant has been reported in an unrelated male proband with CDD [7]. However, the presentation of our patient is not consistent with CDD as he did not experience the persistent decline in functioning that is characteristic of that condition. Thus, the present case represents a potential expansion of the *TRRAP*-associated phenotype, and supports the hypothesis that de novo *TRRAP* mutations can be responsible for clinically diverse neurological conditions.

Shared genetic etiologies and common pathophysiologies have been shown for psychiatric disorders [28], and the TRRAP protein has several predicted binding partners [29] that provide additional evidence for its role in neurological function. Lysine acetyltransferase 2A, encoded by the *KAT2A* gene, is involved in memory formation and consolidation [30]; *Kat2a* −/− mice exhibit decreased hippocampal synaptic plasticity and impaired long-term memory consolidation [30]. Additionally, variants of the predicted binding partner Actin Like 6A, encoded by *ACTL6A*, are linked to intellectual disability syndromes [31].

Currently, with recovery from his depression and psychotic episode, the proband’s function has returned to baseline. He is doing well academically at grade level and although his mood is sensitive to stress, he is again making slow gains socially at school.

Based on our observations, we propose that the *TRRAP* variant p.Arg1986Gln constitutes a new cause of a very early onset psychosis syndrome, characterized by NVLD, OCD, auditory hallucinations, and paranoia in the context of a major depressive episode.

## Additional file

Additional file 1: **Table S1.** All *de novos* detected by exome sequencing This table contains locations and details of all *de novos* detected by exome sequencing in the patient, and evidence for and against pathogenicity. (XLSX 27 kb)

## Abbreviations

aCGH: Array-based comparative genomic hybridization
CDD: Childhood disintegrative disorder
CNV: Copy number variation
HAT: Histone acetyltransferase
LOH: Loss of heterozygosity
NIMH: National Institute of Mental Health
NVLD: Non-verbal learning disability
OCD: Obsessive-compulsive disorder
SNV: Single nucleotide variant
SNVs: Single nucleotide variants
SZ: Schizophrenia
VEOP: Very early onset psychosis
WES: Whole exome sequencing
